# Transcutaneous auricular vagus nerve stimulation induces stabilizing modifications in large-scale functional brain networks: towards understanding the effects of taVNS in subjects with epilepsy

**DOI:** 10.1038/s41598-021-87032-1

**Published:** 2021-04-12

**Authors:** Randi von Wrede, Thorsten Rings, Sophia Schach, Christoph Helmstaedter, Klaus Lehnertz

**Affiliations:** 1 Department of Epileptology, University Hospital Bonn, Venusberg Campus 1, 53127 Bonn, Germany; 2 Helmholtz-Institute for Radiation and Nuclear Physics, University of Bonn, Nussallee 14-16, 53115 Bonn, Germany; 3 Interdisciplinary Center for Complex Systems, University of Bonn, Brühler Straße 7, 53175 Bonn, Germany

**Keywords:** Physics, Statistical physics, thermodynamics and nonlinear dynamics, Complex networks, Neuroscience, Diseases of the nervous system, Epilepsy

## Abstract

Transcutaneous auricular vagus nerve stimulation (taVNS) is a novel non-invasive brain stimulation technique considered as a potential supplementary treatment option for subjects with refractory epilepsy. Its exact mechanism of action is not yet fully understood. We developed an examination schedule to probe for immediate taVNS-induced modifications of large-scale epileptic brain networks and accompanying changes of cognition and behaviour. In this prospective trial, we applied short-term (1 h) taVNS to 14 subjects with epilepsy during a continuous 3-h EEG recording which was embedded in two standardized neuropsychological assessments. From these EEG, we derived evolving epileptic brain networks and tracked important topological, robustness, and stability properties of networks over time. In the majority of investigated subjects, taVNS induced measurable and persisting modifications in network properties that point to a more resilient epileptic brain network without negatively impacting cognition, behaviour, or mood. The stimulation was well tolerated and the usability of the device was rated good. Short-term taVNS has a topology-modifying, robustness- and stability-enhancing immediate effect on large-scale epileptic brain networks. It has no detrimental effects on cognition and behaviour. Translation into clinical practice requires further studies to detail knowledge about the exact mechanisms by which taVNS prevents or inhibits seizures.

## Introduction

Epilepsy is one of the most common neurological disorders and is defined by recurrent epileptic seizures. Although two thirds of affected subjects achieve seizure-freedom with the first two appropriately chosen antiseizure medications (ASM)^1^, the other third requires extensive therapy attempts in order to achieve seizure-freedom or at least an acceptable seizure situation. Even the development of new ASM has not led to a significant improvement of seizure outcome, though tolerability and interaction profile have become more advantageous^2^. Thus, there is a strong need for alternative or complementary treatment options. Vagus nerve stimulation (VNS) is an established method of brain stimulation in several diseases, including epilepsy^3^. Invasive vagus nerve stimulation (iVNS) was first approved as early as in the 1990s. It has been extensively studied and its safety has been demonstrated in more than 20 studies. Its effectiveness is assumed with a responder rate (subjects in whom seizure frequency is reduced by more than 50%) of approximately 50%^4,5^. However, it is an invasive method with need of anaesthesia and surgical risk. Transcutaneous auricular vagus nerve stimulation (taVNS) is a non-invasive external stimulation (of the auricular branch of the vagus nerve) and seems to be an interesting alternative. Good tolerability and effectiveness have been demonstrated for taVNS^6–10^. For both iVNS and taVNS, similar projections of afferent vagus nerve fibres to the nucleus of the solitary tract could be shown^11^ and cerebral activation patterns induced by iVNS and taVNS resemble each other (for overview see^12^).

Knowledge about immediate and longer-lasting VNS-related changes of brain activity is sparse. In contrast to other, locally specific stimulation methods such as deep brain stimulation (DBS)^13^ or responsive neurostimulation (RNS)^14^, it is generally assumed that VNS leads to a rather unspecific, global activation of various brain structures (including thalamus, limbic system, insular cortex)^15,16^. This local unspecificity is also reflected in contradicting findings on the EEG: while some authors report a modification of epileptiform activity^17^, quantitative EEG studies point to opposing phenomena (e.g., synchronisation vs. desynchronisation^16^) as well as to ambiguous changes in relevant EEG frequency bands^18^.

**Figure 1:**
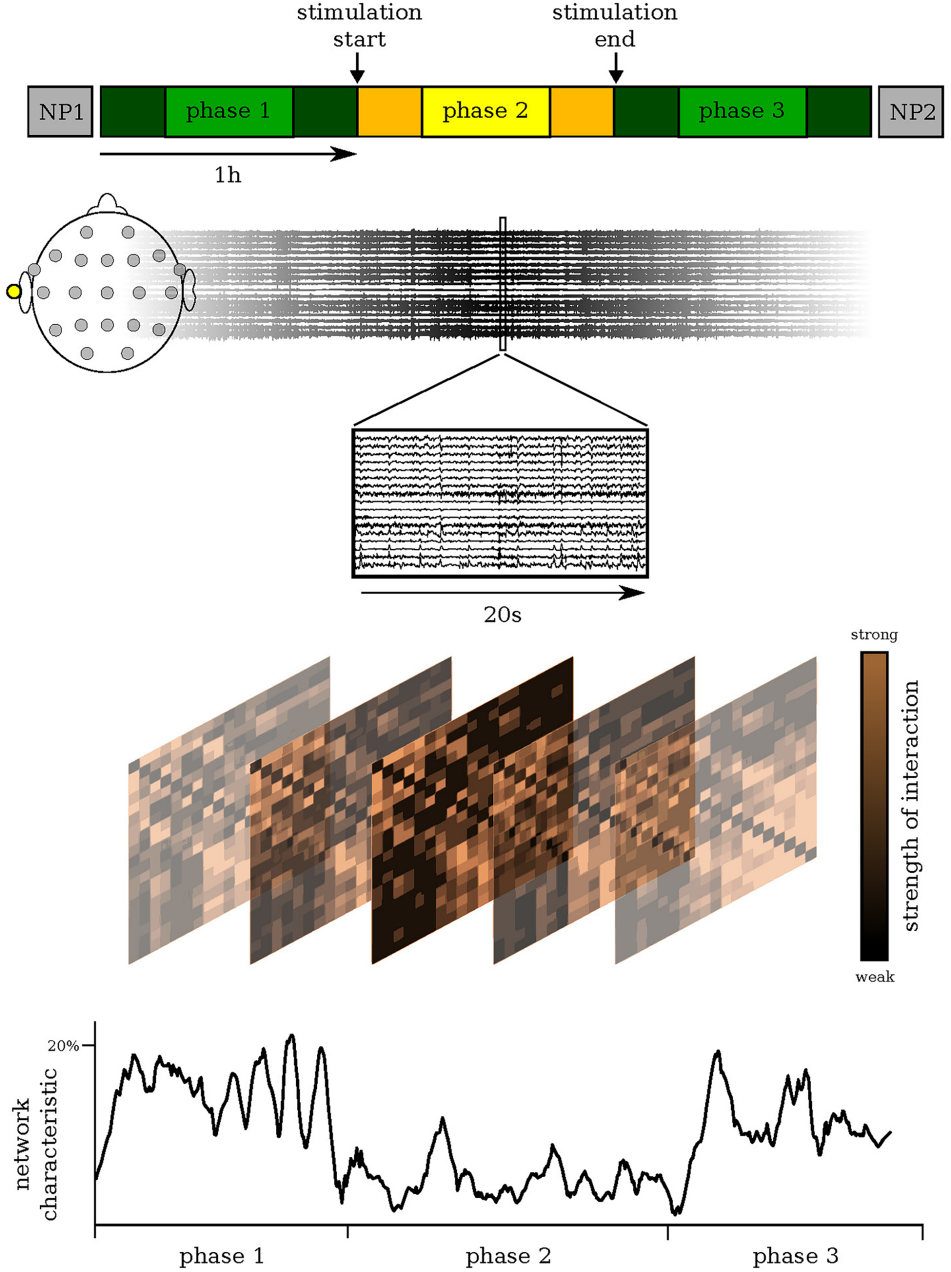
Examination schedule: Probing for taVNS-induced changes in epileptic brain networks. Our examination schedule consisted of a 3-h EEG recording (Methods) that covered a stimulation phase (phase 2; continuous stimulation of the left cymba conchae) and a pre- and post-stimulation phase (phase 1 and phase 3, resp.). In our analyses, we neglected data from the first and last 15 min of each phase (darker colours) in order to remove possible transient effects. The EEG recording was preceded and followed by a standardized neuropsychological assessment (NP1 and NP2, resp. 30 min; Methods). We derived evolving epileptic brain networks from the EEG recording using a sliding-window approach (Methods), assessed important global characteristics of each network (Methods), and tracked their changes over time.

We hypothesized that the impact of the global, apparently unspecific activation can be suitably assessed with a global analysis approach which makes use of the EEG derived so-called evolving functional brain networks^19,20^. The powerful mathematical framework of network theory provides means to determine important network characteristics such as their topological, stability, and robustness properties. Tracking network characteristics over time would allow one to identify and delineate stimulation-related changes of EEG activity. Accompanying such an investigation with an examination of cognitive functions may provide important insights into their possible relationships with the aforementioned network characteristics^21^ and could help to improve understanding of whether and how VNS may impact cognition^22,23^. We tested this hypothesis by investigating whether short-term taVNS induces measurable immediate modifications of functional brain network in subjects with epilepsy and whether modifications are accompanied by changes of cognition and behaviour (see Fig. 1).

## Results

### Stimulation-related modifications of evolving epileptic brain networks

Evolving epileptic brain networks are functional networks^19^ that can be derived from EEG recordings by associating network vertices with brain regions sampled by electrode contacts and network edges with the time-varying estimates of the strength of interactions between pairs of brain regions^20^ (Methods). We derived such evolving, fully connected and weighted networks from a time-resolved synchronisation analysis of the 3-h EEG recording, used various measures (Methods) to assess important characteristics of each network and tracked their changes over time. In order to characterise the network’s global topological properties, we estimated its average shortest path length *L* and its average clustering coefficient *C*. In addition, we assessed the network’s stability and robustness properties by estimating its synchronisability *S* and its assortativity *A*. The average shortest path length characterises the network’s functional integration; the lower *L*, the more integrated is the network. The average clustering coefficient characterises the network’s functional segregation; the lower *C*, the more segregated is the network. Synchronisability assesses the network’s propensity (or vulnerability) to get synchronised by an admissible input activation: the lower *S*, the more easily can the synchronised state be perturbed. Assortativity assesses the tendency of edges to connect vertices with similar or equal properties. If edges preferentially connect vertices of similar (dissimilar) property, such networks are called assortative (disassortative). Disassortative networks are more vulnerable to perturbations and appear to be easier to synchronise than assortative networks. The latter show a stronger tendency to disintegrate into different groups than disassortative networks.

**Figure 2:**
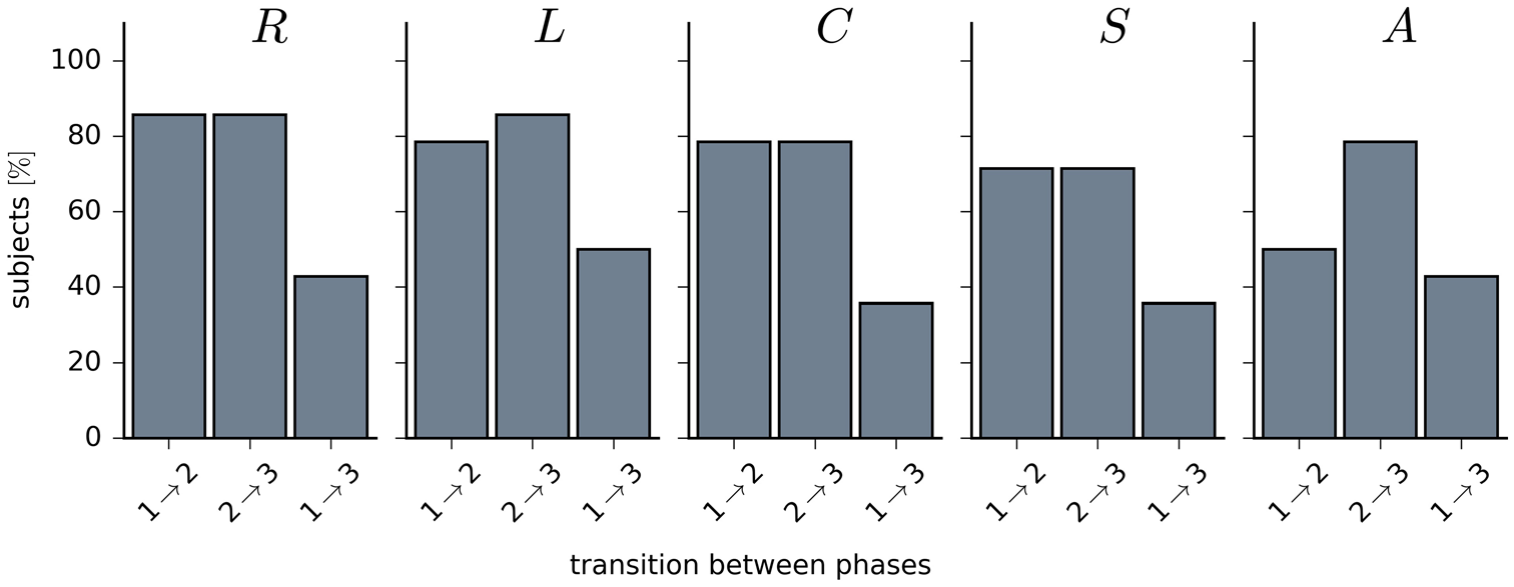
Percentage of subjects for which taVNS led to significant differences (Methods) between networks characteristics from phases 1, 2, and 3; global synchronization level *R*, average shortest path length *L*, average clustering coefficient *C*, synchronisability *S*, and assortativity *A*.

In the majority of subjects (Fig. 2), taVNS led to immediate, stimulation-related alterations in the overall strength of functional interactions (global synchronisation level *R*) in epileptic brain networks. Their average shortest path length *L*, average clustering coefficient *C*, synchronisability *S*, and assortativity *A* were seen to be modified in a similar number of subjects. Interestingly, taVNS appeared to have a persistent effect in about 30–50% of subjects, as seen with most network characteristics (for those subjects, for which we achieved significant differences between phases ($p<0.05$ after Bonferroni correction, Mann–Whitney U values ranged between 173 and 3187 (phase $1 \rightarrow 2$), between 23 and 3231 (phase $2 \rightarrow 3$) and between 585 and 3301 (phase $1 \rightarrow 3$); ranges are reported for all network characteristics; the number of degrees amounted to 90 for each phase).

**Figure 3:**
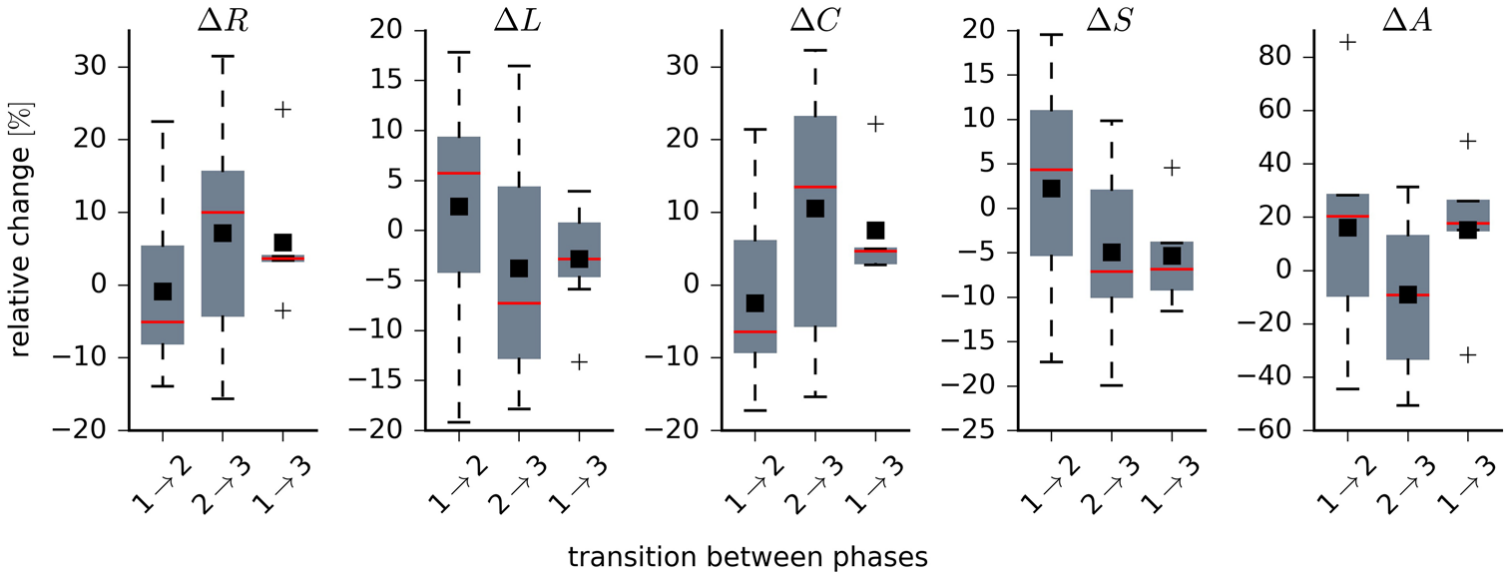
Distributions of taVNS-related alterations in network characteristics. Boxplots of relative changes $\Delta$ in network characteristics. Relative changes calculated as $\Delta =(M_l-M_k)/M_k$, where $M_k$ and $M_l$ denote placeholders for the temporal means of the respective characteristics from phase *k* and phase *l* (global synchronization level *R*, average shortest path length *L*, average clustering coefficient *C*, synchronisability *S*, and assortativity *A*). During phase 1, network characteristics attained the following values: $R = 0.31 \pm 0.02$, $L = 3.43 \pm 0.29$, $C = 3.33 \pm 0.02$, $S = 3.15 \pm 0.49$, and $A = 0.37 \pm 0.15$. Bottom and top of a box are the first and third quartiles, and the red band and the black square are the median and the mean of the distribution. The ends of the whiskers represent the interquartile range of the data. Outliers are marked by a + sign.

We provide a more detailed picture of stimulation-related alterations of network characteristics in Fig. 3, where we plot the distributions of their relative changes for networks transiting between the different phases. The global synchronisation level *R* slightly decreased from the pre-stimulation to the stimulation phase (desynchronisation; phase $1 \rightarrow 2$: −5%; we report the median values in the following) but it increased when networks transit from the stimulation to the post-stimulation phase (re-synchronisation; phase $2 \rightarrow 3$: 10%). We observed only slight differences between the pre- and post-stimulation phase (phase $1 \rightarrow 3$: 4%). Together with the high interindividual variability, these findings partly confirm previous observations with long-term iVNS^24,25^ or immediate iVNS^26^.

For the average shortest path length *L*, we attained a similar though inverted patterning (which is to be expected given the definition of a path length in a weighted network): a slight increase of *L* from the pre-stimulation to the stimulation phase (phase $1 \rightarrow 2$: 6%), a slight decrease from the stimulation to the post-stimulation phase (phase $2 \rightarrow 3$: −7%), and slight undershoot effect when comparing the pre- and post-stimulation phase (phase $1 \rightarrow 3$: −3%).

We can derive similar indications with changes of the average clustering coefficient *C*, for which we observed a patterning that compares to the one seen for the global synchronisation level, although changes were slightly more pronounced (phase $1 \rightarrow 2$: −6%; phase $2 \rightarrow 3$: 14%; phase $1 \rightarrow 3$: 5%).

Synchronisability *S* slightly increased from the pre-stimulation to the stimulation phase (phase $1 \rightarrow 2$: 4%) and it decreased when networks transit from the stimulation back to the post-stimulation phase (phase $2 \rightarrow 3$: −7%). A similar decrease was observed when comparing the pre- and post-stimulation phase (phase $1 \rightarrow 3$: −7%).

Interestingly, we obtained strongest indications for a preventive effect of taVNS with changes in the networks’ assortativity *A*. Already during the pre-stimulation phase, epileptic brain networks were seen to be assortative ($A = 0.37 \pm 0.15$). taVNS even increased their assortativity (phase $1 \rightarrow 2$: 20%). Although networks experienced a slight decrease of their robustness when transiting from the stimulation back to the post-stimulation phase (phase $2 \rightarrow 3$: −7%), the comparably strong increase seen between the pre- and post-stimulation phase (phase $1 \rightarrow 3$: 18%) would point to an enduring robustness-enhancing effect of taVNS.

### Stimulation-related modifications of cognition and behaviour

Prior to stimulation, 35% of subjects presented with impaired executive functions. Mild or severe impairment in verbal memory was seen in 82%, and a relevant depressive symptomatology in 43% of subjects. After stimulation, we observed in two subjects a significant intraindividual improvement of executive functions. All other cognition-related variables remained unaffected in these and the other subjects (Mann Whitney U values ranged between 64 and 98 for the different domains; the number of degrees of freedom amounted to 14; n.s.). No significant self-perceived changes in the evaluated domains cognition, behaviour and physiological symptoms were observed; one subject reported an improvement in anxiety after taVNS. There were no significant relationships between neuropsychological variables and characteristics of epileptic brain networks.

### Evaluation of the device: side effects and usability

No local side effects were complained or detected by clinical check-up. All subjects rated the handling of the device as good or very good. 86% felt that the continuation of their activities was not affected by the stimulation. The majority rated the wearing comfort as good or very good (79%). However, some subjects stated that the device is rather poorly suited for long-term use during the day (43%) or repeated use within one day (29%).

## Discussion

With our prospective trial, we investigated whether short-term transcutaneous auricular vagus nerve stimulation (taVNS) induces measurable immediate modifications of functional brain networks in fourteen subjects with epilepsy and whether modifications are accompanied by changes of cognition and behaviour. Our findings reveal that taVNS has stabilising effects on networks in the majority investigated subjects and these effects persist in up to 50% of subjects. In contrast, cognition and behaviour are not affected by the stimulation.

The stimulation-related alterations seen for network characteristics average shortest path length and clustering coefficient indicate that taVNS modifies the network’s topological organisation, which is reflected in a more integrated and less segregated network. Similar findings could be achieved only recently also with long-term invasive vagus nerve stimulation^27^. In addition to modifications of network topology, short-term taVNS can enhance stability and robustness of epileptic brain networks. The alterations seen for synchronisability indicate an increase of the network’s stability against perturbations, i.e., a more resilient brain network. Moreover, the observed similar decrease in synchronisability when comparing the pre- and post-stimulation phases would point to an enduring stabilising effect of taVNS. Interestingly, we obtained strongest indications for a preventive effect of taVNS with changes in the networks’ assortativity. Already prior to stimulation, epileptic brain networks were seen to be assortative, which confirms previous observations^28,29^, and taVNS even increased their assortativity. Although networks experienced a slight decrease of their robustness when transiting from the stimulation back to the post-stimulation phase, the comparably strong increase seen between the pre- and post-stimulation phase would point to an enduring robustness-enhancing effect of taVNS.

There were no detrimental effects of taVNS on cognition and behaviour in our subjects with epilepsy. Similar observations were made recently in healthy subjects^30^, even when stimulating the brain during the memory consolidation phase. However, findings need to be taken with care, given that research into the impact of taVNS on cognition is still in its infancy^31,32^. Previous studies revealed that long-term iVNS can enhance recognition memory in subjects with epilepsy in comparison to sham stimulation and depending on stimulation intensity^33^. Detrimental effects, however, were reported for acute high-intensity iVNS on figural memory but not on verbal memory in subjects with epilepsy^22^. Future studies would need to further elucidate the influence of taVNS on cognition and behaviour.

Short-term taVNS was well tolerated by our subjects with epilepsy, and no local side effects occurred. These results are in par with expected results from long-term studies^34^. The usability of the device was rated good and very good in terms of handling, management, comfort, and possibility of continuation of one’s activities. However, rating for suitability for long-term or repeated use was viewed critically by some subjects. Complaints about the duration of a daily stimulation of 4 h were given by subjects with epilepsy in treatment settings before and recently led to an evaluation of the effects of reduced stimulation times^9^.

Brain stimulation is a rapidly evolving field and is considered as a supplementary treatment option for subjects with refractory epilepsy. Invasive VNS is accompanied with perioperative risks involved with device implantation and is thus limited to the treatment of more severe, drug-resistant cases. taVNS is a non-invasive brain stimulation technique and clinical data about efficacy and tolerability indicate this approach to be an interesting alternative. Nevertheless, we still lack detailed knowledge about the exact mechanisms—from the molecular^35^ to the brain level and to other organs (e.g. heart^36^)—by which taVNS prevents or inhibits seizures which currently hinders the translation into clinical practice^37^. Our findings point to a topology-modifying, robustness- and stability-enhancing immediate effect of short-term taVNS on large-scale epileptic brain networks. At least on the time scale considered in our study (few hours), these network modifications did not impact on the investigated variables of cognition and behaviour. Our approach thus opens new perspectives towards improving our understanding of the dynamics of large-scale epileptic brain networks as well as towards deciphering the mechanism of action of taVNS.

Future studies should investigate the impact of long-term transcutaneous auricular vagus nerve stimulation on brain networks as well as long-term effects of the stimulation to deepen understanding of the mechanism of action and the potential efficacy of taVNS. By the same token, future studies should also investigate the impact of stimulation on local and/or medium-scale properties of epileptic brain networks (such as centralities of vertices and edges, cores, motifs, or community structures) as this could help in optimizing stimulation parameters which are currently selected rather heuristically. Comparing the effects of iVNS and taVNS using the same study design could reveal similarities and differences of these stimulation approaches with regard to large-scale epileptic networks. And finally, evaluation of subjects with different epilepsy syndromes and different severities could help translating this brain stimulation approach into clinical use.

There are some limitations of our prospective investigations. Since we avoided as much confounders on the EEG-evaluation as possible (e.g. by activation methods, change of ASM, seizures before study), we generated a high exclusion rate that led to a small number and higher heterogenicity of investigated subjects with epilepsy. A larger group size as well as more homogeneous groups could be interesting. The device we used for taVNS has non-adjustable stimulation parameters. However, in iVNS, adjusting parameters individually is not only crucial for an effective treatment of epilepsy^38,39^, but might also impact on topological and robustness properties of epileptic brain networks. Evaluating the impact of varying stimulation parameters could contribute to the understanding of the mechanism of actions of taVNS and, in the long run, help to optimize its clinical use.

To conclude, short-term taVNS has a topology-modifying, robustness- and stability-enhancing immediate effect on large-scale epileptic brain networks. It has no detrimental effects on cognition and behaviour and was well tolerated by our subjects with epilepsy. There are similarities between taVNS and iVNS that emphasise the necessity of further research on taVNS as the less complicated way of brain stimulation via the vagus nerve.

## Methods

### Subjects

Between March 25 and September 19 of 2020, 472 subjects were admitted to our ward and were screened for suitability for our study. Exclusion criteria were unclear diagnosis, progressive disease, previous resective brain surgery, actual or previous vagus nerve stimulation or deep brain stimulation, insufficient German language capability, mental disability and incompetence to follow instructions. Inclusion criteria were clinical necessity for long-term video EEG-recording and proven diagnosis of epilepsy. Of the 36 eligible subjects, 22 declined participation. Fourteen subjects signed informed consent after being provided with written information and being given the opportunity to ask further questions; these subjects were included in the study. The study protocol had been approved by the ethics committee of the University of Bonn before the study has started. All experiments were performed in accordance with relevant guidelines and regulations.

**Table 1:** Patient demographics. Dur.: duration of disease in years; lat. = lateralization; loc. = localisation; hand. = handedness; drug res. = drug resistance according to ILAE^40^; ASM = antiseizure medication; LEV = levetiracetam, LTG = lamotrigine; LCM = lacosamide; TPM = topiramate; BRV = brivaracetam; VPA =valproate; PB = phenobarbital, OXC = oxcarbazepine, CBZ = carbamazepine; ZON = zonisamide; stim. = stimulus intensity in mA.

	Sex	Age	Dur.	Lat.	Loc.	Hand.	MRI lesion	Drug res.	ASM	Stim.
1	f	50	1	Right	Insula	Right	Yes	No	LEV	3.0
4	f	19	0	Left	Frontal	Right	No	No		0.9
5	m	18	0	Right	Temporal	Ambidexter	No	No	LEV	0.9
6	m	25	1	Unknown	Unknown	Right	No	No		3.5
7	f	22	7	Right	Frontal	Right	No	Yes	LCM	0.6
9	f	55	4	Right	Temporal	Right	No	Yes	LEV, TPM	3.0
11	f	24	12	Bilateral	Temporal	Right	No	Yes	BRV, LTG, LCM	3.0
12	f	70	60	Right	Temporal	Right	Yes	Yes	LTG, VPA, PB	0.9
14	m	71	1	Left	Temporal	Right	No	No	LEV	1.4
15	m	26	19	Left	Frontal	Right	Yes	Yes	LEV, LTG, VPA, OXC	1.9
17	f	25	5	Right	Frontal	Right	No	Yes	CBZ	2.9
18	m	77	2	Left	Temporal	Right	No	No	LEV	1.6
19	f	53	34	Left	Temporal	Right	No	Yes	LTG, ZON	1.9
20	m	40	17	Right	Temporal	Right	No	Yes	LEV, OXC	2.7

Fourteen subjects with epilepsy (8 females; age 18–77 years, median 41 years; Table 1) were included in the study. Eight subjects had a drug-resistant epilepsy according to the definition of the International League against Epilepsy^40^. We applied taVNS with individualized stimulation intensities (range: 0.6–3.5 mA. mean 2.0, SD $\pm 1.0$, Methods) for 1 h in the early afternoon while subjects underwent a continuous 3-h EEG recording (see Fig. 1). No activation methods (such as change in ASM, hyperventilation or sleep deprivation) were applied at least 24 h before stimulation. The EEG recording was preceded and followed by a standardized neuropsychological assessment which involved measures of executive functions, verbal memory, mood, and the rating of subjective changes of the subjects’ cognitive, psychiatric and somatic condition. To reduce potential practice effects, parallel test versions were applied for examining executive functions and verbal memory. No side effects were reported or observed.

### Transcutaneous auricular vagus nerve stimulation

Stimulation was carried out with two hemispheric titanium electrodes of a NEMOS device (tVNS Technologies GmbH, Erlangen, Germany) fitted in the left cymba conchae and using a common set of non-adjustable parameters (biphasic signal form, impulse duration 20 s, impulse pause 30 s, impulse frequency 25 Hz). Intensity of stimulation was adjusted individually and was raised slowly until the subject noticed a “tingling”, but no pain.

### Details of neuropsychological assessment

*Attention and executive functions*. The EpiTrack 3rd edition^41^ is a screening tool consisting of six subtests assessing response inhibition, visuo-motor speed, mental flexibility, visuo-motor planning, verbal fluency, and verbal working memory. It can be completed in 15 min. The performance in each subtest results in an age-corrected total score with a maximum score of 49 points (after age-correction). Mild impairment is reflected by a total score in the range of 29 to 31, the cut-off score for severe impairment is $\le 28$ points ($> 2$ SD below the normative sample). A significant intraindividual change in the total scores between two assessments is indicated by a gain of $\ge 4$ points or the loss of $\ge 3$ points.

*Verbal memory*. Verbal memory was assessed using a short version of the Verbal Learning and Memory Test (VLMT^42^) which is the German adaptation of the Rey Auditory Verbal Learning Test (RAVLT). The shortened VLMT version includes two consecutive trials of word list learning (15 words) with immediate free recall. After the two learning trials, the EpiTrack was performed, followed by the delayed free recall of the word list. Thus, the EpiTrack provided a distraction for memory testing. Age-correction was based on normative data of 383 healthy subjects. Scores for learning, memory and loss over time were transformed into a scale ranging from 1 to 7 according to the normative sample and converted into a total memory score ranging from 3 to 21. After age correction, total memory scores from 14 to 18 are rated as normal, scores $>18$ as above average, scores from 11 to 13 as mild impairment, and scores of $\le 10$ are considered a significant impairment. A significant change is indicated by a gain of $>3$ points or a loss of $>5$ points.

*Mood/Depression.* The Neurological Disorders Depression Inventory for Epilepsy (NDDI-E^43^) is a brief self-report questionnaire used as a screening tool for detecting depression in people with epilepsy. This 6-item screening instrument specifically focuses on symptoms of depression that cannot be explained by adverse effects of antiseizure medication. All items are rated on a four-tiered scale (1—never, 2—rarely, 3—sometimes, 4—always or often). A total score above 15 indicates a relevant depressive symptomatology.

*Subjective measures*. A modified version of the Adverse Events Profile was used before and after stimulation to assess self-perceived changes in three domains: (1) cognition (vigilance, energy, psychomotor speed, attention/ability to concentrate, fluent speech, verbal comprehension, word finding, remote memory), (2) behaviour (depression, anxiety, aggression, restlessness), (3) physiological symptoms (dizziness, drowsiness, nervousness, tremor, headache, nausea, dermatological symptoms, vision problems/double vision). Subjects were asked to rate the presence and severity of impairments on a four-tiered scale ranging from very good (0) to very bad (3). Total scores for each domain were calculated.

*Questionnaire on the evaluation of the device*. Seven ordinal questions were asked concerning handling, possibility to continue activities while using the device, feeling while using the device, comfort, suitability for long-term and repeated use.

### EEG recordings and data pre-processing

We recorded electroencephalograms (EEG) from 19 electrode sites according to the 10–20 system and Cz served as physical reference. EEG data were sampled at 256 Hz using a 16 bit analogue-to-digital converter and were band-pass filtered offline between 1–45 Hz (4th order Butterworth characteristic). Additionally, a notch filter (3rd order) was used to suppress contributions at the line frequency (50 Hz). We visually inspected all recordings for strong artefacts such as subject movements, amplifier saturation, or stimulation artefacts. Such data were excluded from further analyses.

We used a sliding-window approach^44–46^ to calculate a synchronisation index $r_{ij}$ (mean phase coherence^47^) between phase time series (derived adaptively with Hilbert transform^48^) from all pairs of brain regions (*i*, *j*) sampled by the EEG electrodes. Non-overlapping windows had duration of 20 s (5120 data points), which represents a compromise between the required statistical accuracy for the calculation of $r_{ij}$ and approximate stationarity within a window length.

The synchronisation index serves as an indicator for the strength of functional interactions in the epileptic brain network^45^ and is confined to the unit interval: $r_{ij} = 1$ indicates fully phase-synchronised brain regions and $r_{ij} = 0$ indexes no phase synchronisation. For subsequent analyses, we associated the sampled brain regions with network vertices and the calculated phase synchronisation indices between any pair of vertices with network edges. This resulted in a time-dependent sequence of weighted and fully connected brain networks.

### Network characteristics

In addition to global synchronisation level *R* (mean over all non-redundant pairwise synchronisation indices), we assessed four relevant global characteristics for each network that we derived from the time-resolved synchronisation analysis of the 3-h EEG recording prior to (phase 1), during (phase 2), and after taVNS (phase 3): average shortest path length *L*, average clustering coefficient *C*, synchronisability *S*, and assortativity *A*. In order to remove possible transient effects, we neglected data from the first and last 15 min of each phase.

The average shortest path length *L* is defined as the average number of steps along the shortest paths for all possible pairs of network vertices. For our weighted networks, we defined the ‘length’ of a path between a pair of vertices as the inverse of the weight of the edge that connects the vertices^20^ and used an algorithm proposed by Dijkstra^49^ to compute *L*. The clustering coefficient is a measure of the degree to which vertices in a network tend to cluster together. We made use of a definition of the clustering coefficient in a weighted network^50^ and calculated the average clustering coefficient *C* as the mean of clustering coefficients computed for all vertices. Synchronisability *S* is a measure of the stability of the network’s synchronised state^51,52^. We computed *S* from the ratio of the largest and smallest non-vanishing eigenvalue that we calculated for the network’s Laplacian^53^. To assess assortativity *A* of the networks^54^, we estimated the Pearson correlation coefficient between the degrees of vertices at both ends of an edge^55^. To this end, we derived a connected binary network from the weighted network by thresholding thereby requiring a constant edge density. *A* is confined to the interval [−1, 1] by definition. Positive (negative) values of *A* indicate an assortative (disassortative) network.

### Statistical analyses

Differences between network characteristics from the three phases (phase 1: pre-stimulation; phase 2: during stimulation; phase 3: post-stimulation; see Fig. 1) were investigated on a per-subject basis using the Mann–Whitney U-test (phase 1 vs. phase 2, phase 1 vs. phase 3, and phase 2 vs. phase 3). For downstream network analyses, we only considered data from subjects for whom we attained significant differences after Bonferroni correction ($p < 0.05$). Group level (all subjects) differences between neuropsychological variables from the phases prior to and after the EEG recording (NP1 vs. NP2; see Fig. 1) were investigated using the Mann–Whitney U-test ($p < 0.05$). Finally, we probed for possible relationships between the aforementioned changes in neuropsychological variables and (a) network characteristics (temporal means) from the three phases and (b) relative changes of network characteristics between the three phases (relative changes calculated as $\Delta =(M_l-M_k)/M_k$, where $M_k$ and $M_l$ denote placeholders for the temporal means of the respective characteristics from phase *k* and phase *l*). Relationships were deemed significant after Bonferroni correction (Pearson correlation coefficient; $p < 0.05$).

## Data Availability

The data that support the findings of this study are available from the corresponding author upon reasonable request. The data are not publicly available as they contain information that could compromise the privacy of research participants.
